# Significant response to pembrolizumab plus lenvatinib in Epstein–Barr-virus-associated intrahepatic cholangiocarcinoma: a case report

**DOI:** 10.1080/15384047.2024.2338644

**Published:** 2024-04-22

**Authors:** Lisha Li, Dandan Yu, Jinru Yang, Fangyuan Zhang, Dejun Zhang, Zhenyu Lin, Menglan Zhai, Jing Wang, Tao Zhang, Lei Zhao

**Affiliations:** Cancer Center, Institute of Radiation Oncology, Hubei Key Laboratory of Precision Radiation Oncology, Union Hospital, Tongji Medical College, Huazhong University of Science and Technology, Wuhan, China

**Keywords:** Pembrolizumab, lenvatinib, Epstein–Barr virus, intrahepatic cholangiocarcinoma

## Abstract

**Background:**

The prognosis for advanced intrahepatic cholangiocarcinoma (iCCA) is poor, and there remains an urgent need to develop efficient systemic therapy. The efficacy of Pembrolizumab immunotherapy combined with lenvatinibin in iCCA is still unclear. The role of Epstein–Barr-virus (EBV) as a biomarker in iCCA for response to immunotherapy needs further exploration.

**Case presentation:**

We report a case of a 60-year-old female with EBV-associated advanced iCCA (EBVaiCCA) who progressed after first-line therapy. She accomplished an available response to the combination therapy of pembrolizumab with lenvatinib, with overall survival of 20 months.

**Conclusions:**

As far as we know, this is the first case report about the application of Pembrolizumab with lenvatinib for EBVaiCCA patients. This case indicates that the combination of immunotherapy and antiangiogenic therapy provides a glimmer of hope for advanced EBVaiCCA patients.

## Background

Cholangiocarcinoma (CCA) is heterogeneous and is a highly lethal, epithelial cell malignancy that includes intrahepatic CCA (iCCA), perihilar CCA (pCCA), or distal CCA (dCCA) according to the location.^1,2^ iCCA contributes to about 20% of primary liver cancer whose incidence is second only to that of hepatocellular carcinoma (HCC). It has been on the rise globally in the past year with high aggressiveness, malignant biological behavior, and poor outcome.^3^ Therefore, the survival in case of iCCA is very short (typically ≤1 year following diagnosis), and the only possible sanative therapy option is radical resection. Even following surgery, the recurrence rate is very high.^4^ For iCCA patients with locoregional disease, the most promising therapies contained transarterial radioembolization (TARE), hepatic artery infusion (HAI), transarterial chemoembolization (TACE), and radiofrequency ablation (RFA) ,^5–8^ However, iCCA is continually diagnosed when advanced and unresectable. Therefore, systemic therapies are the feasible therapy option for metastatic iCCA patients. The Advanced Biliary Cancer (ABC)-02 trial, a phase III, provided the combination of cisplatin plus gemcitabine (CisGem) to be the standard first-line care over the last 13 years, and the median overall survival is <1 year. Therefore, there remains an urgent need to develop efficient systemic therapy for advanced iCCA. A wide range of clinical trials are currently finding the niche of targeted treatments and immunotherapy in advanced CCA.

Since the US Food and Drug Administration (FDA) approved the anti-PD-L1 agent pembrolizumab for the therapy of any dMMR or MSI-H solid tumors in 2017,^9^ immune checkpoint inhibitors (ICIs) have reshaped the medical landscape of several malignancies.^10^ However, the Keynote-028 trial and the Keynote-158 trial had proved that anti-PD-L1 agent pembrolizumab as monotherapy for CCA is not encouraging.^10^ A lot of preclinical data suggested combining tyrosine kinase inhibitors (TKIs) with ICIs, which could amplify the activity of the immune system. Combining lenvatinib plus pembrolizumab (LEP) has indicated a considerably activated antitumor reaction in a lot of malignancies.^11^ As Lin et al. reported, LEP is a hopeful replacement as second-line therapy for advanced CCA patients.^12^

Recently, people found that high microsatellite instability (MSI-H), increased tumor mutational burden (TMB), deficiency in mismatch repair (dMMR) proteins, PD-L1 expression, and Epstein–Barr virus (EBV) are considered biomarkers for the answer to immunotherapy. Some of them have shown promise in predicting the benefit of PD-1/PD-L1 inhibitor in a lot of malignancies.^10^ However, the role of these biomarkers has not been systematically assessed in advanced iCCA patients. The optimal treatment conditions and efficiency of LEP in iCCA patients remain unknown. A wide number of well-designed clinical trials are still warranted to explore how biomarkers could improve therapy selection for advanced iCCA patients.^10^ EBV is one of the eight human herpesviruses and is related to the development of cancer. EBV-associated cancer accounts for about 1.5% of all cases of cancer and might contribute to immune evasion.^13^ Besides, EBV-associated ICC accounts for about 3.3% to 6.6%.^14,15^ Up to now, EBV has gradually become one of the potential indicators of immunotherapy for some malignancies like gastric cancer. However, the role of EBV in the immunotherapy of iCCA is still under probe.^16^

In the research, we report a case of a 60-year-old Asian female with EBV-associated advanced iCCA. She accomplished an effective answer to LEP therapy, and her overall survival was 20 months.

## Case presentation

**Figure 1.**
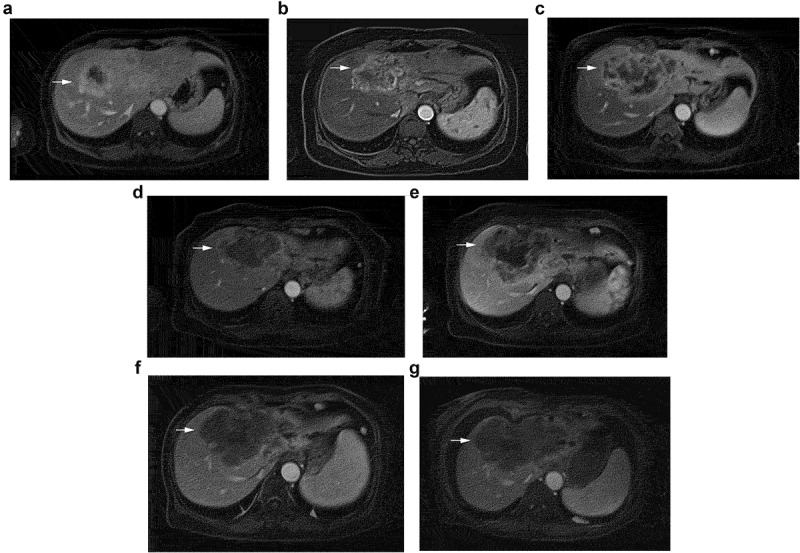
The change of enhanced computed tomography of liver.

**Figure 2.**
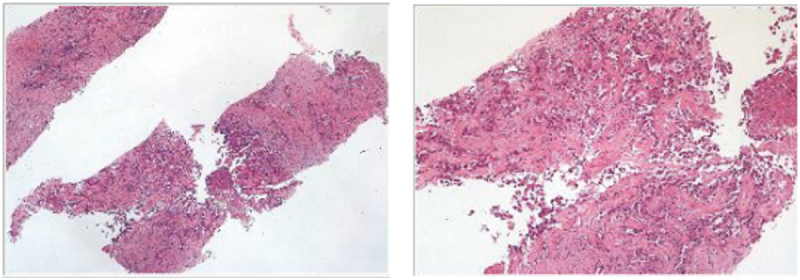
Histological pictures of liver biopsy.

A 60-year-old female was referred to the hospital with a three-month history of abdominal pain occasionally. The enhanced computed tomography (CT) revealed multiple intrahepatic masses with liver (Figure 1(a)) and retroperitoneal lymph node metastasis. Later, she went to the hospital and positron emission tomography-computed tomography (PET-CT) scans revealed HCC with multiple lymph nodes and bone metastases. Additionally, a liver biopsy was performed and histological considerations suggested poorly differentiated adenocarcinoma of the liver, and histological pictures are shown in Figure 2. Immunohistochemical results demonstrated CK7(+), CK19(+), CDX-2(-), GATA-3(-), Villin(-), TTF-1(-), NapsinA(-), and GCDFP-15(-). The patient was the conventional type (CT) of ICC. Besides, the results of next-generation sequencing (NGS) showed the MSI status was stable. The TMB was determined to be 7.1 mutations/Mb which were described as intermediate (6–19 mutations/Mb). KRAS and ATM gene mutations, which were positive immunotherapy efficacy-related variants, were detected in this case. Besides, five HLA subtypes and four TNB were detected. The results also showed three clinically significant mutations, such as KRAS gene p.Gly12Asp.p.Gly138Arg mutation; EGFR exon 20 p.Pro772_His773insVal insertion mutation; PIK3CA gene p.Glu545Lys mutation. Importantly, according to pathohistological analysis, the tumor was found to be positive for EBER. The patient did not have HBV (Hepatitis B Virus) or HCV (Hepatitis C Virus). Based on the patient’s medical history and examination results, the patient was diagnosed with iCCA with multiple lymph nodes and bone metastases, clinically staged as IV which was not qualified for surgery.

**Figure 3.**
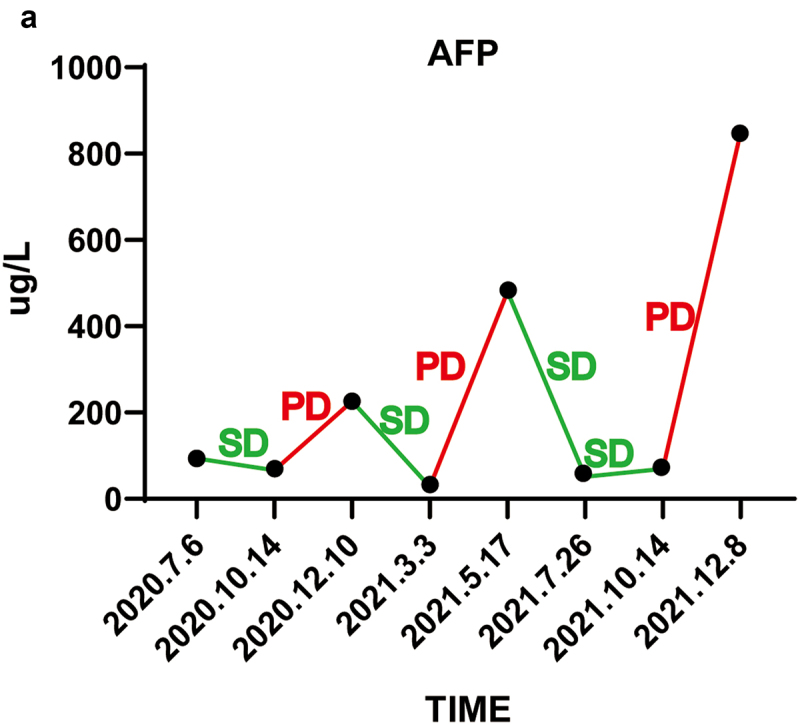
The change of alpha-fetoprotein (AFP).

**Figure 4.**
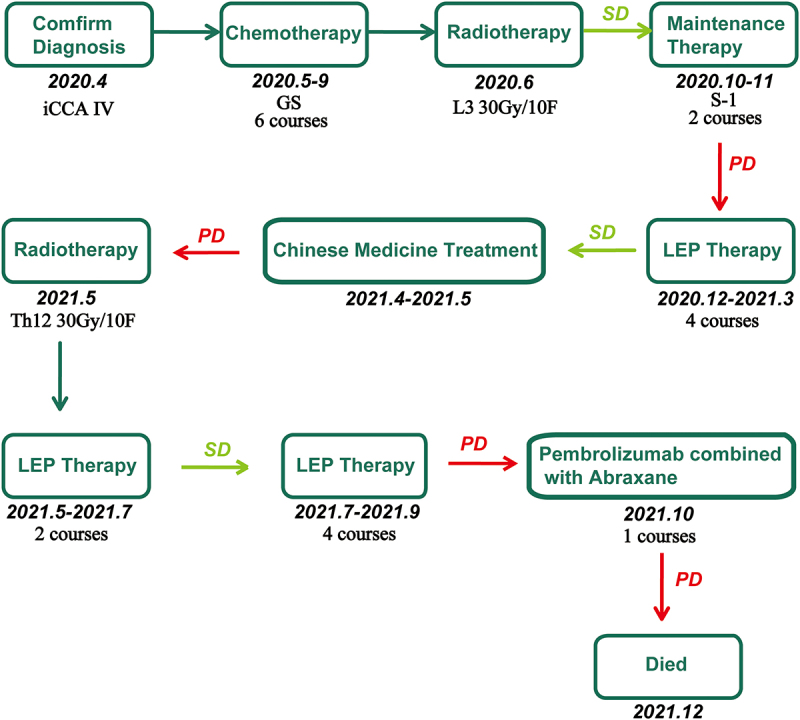
The timeline summarizing the main events of this case report.

The patient received six courses of chemotherapy (intravenous Gemcitabine 1000 mg/m^2^ per day on day 1 and day 8, and S-1 twice a day from day 1 to day 14) and one course of radiotherapy of L3 centrum. An imaging evaluation (Figure 1(b)) suggested stable disease assessed by RECIST 1.1. Then, she received two courses of S-1, and imaging evaluation revealed progressive disease (Figure 1(c)). The patient consequently received four courses of immunotherapy plus targeted therapy (intravenous Pembrolizumab 200 mg three weeks at a time and Lenvatinib 8 mg per day), and imaging evaluation suggested stable disease (Figure 1(d)). After that, she was given LEP once again, and then given Chinese medicine treatment (specifically unknown). However, imaging evaluation suggested progressive disease which might be caused by interruption of treatment (Figure 1(e)). Therefore, she continued receiving radiotherapy combined with LEP, with imaging evaluation suggesting stable disease (Figure 1(f)). The patient could tolerate LEP therapy and did not experience an adverse event. After that, she was given four courses of LEP until imaging evaluation suggested progressive disease with seroperitoneum (Figure 1(g)). The change in alpha-fetoprotein (AFP) is shown in Figure 3. Then, she received one-course Pembrolizumab combined with Abraxane (intravenous Pembrolizumab 200 mg and Abraxane 125 mg/m^2^ per day on day 1 and day 8 for 3 weeks at a time) but had to be interrupted due to poor performance status. She finally died on December 29, 2021, and her overall survival is about 20 months. A timeline summarizing the main events of this case report is shown in Figure 4.

## Discussion and conclusions

In this case, we reported a female patient with IV-stage EBV-associated iCCA (EBVaiCCA) who progressed after first-line therapies but showed a durable response to the combination of LEP therapy. This case showed that prolonging the length of survival can be achieved in a patient with advanced iCCA who received LEP therapy.

Currently, surgery remains the main therapy in the early stages of CCA, but only about 10% to 40% of CCA patients have the chance to receive surgery and the median 5-year survival is less than 50%.^17^ In retrospective studies, various locoregional types of therapy such as TACE and TARE have been reported to improve survival in cases of CCA patients.^18,19^ Besides, the hepatic arterial infusion has also been shown to be of benefit in advanced CCA patients but might lead to more complications.^20–22^ In addition, intraductal radiofrequency ablation and photodynamic therapy can also improve survival in advanced CCA patients.^23,24^ Systemic chemotherapy is the mainstay palliative therapy modality for advanced CCA patients. The ABC-01 trial showed an improvement in PFS in CCA patients who received CisGem.^25^ After that, phase III randomized ABC-02 trial and the randomized phase II BT22 provided CisGem to be the standard first-line care.^26,27^ Besides, according to a nonrandomized phase II trial, the combination of gemcitabine plus oxaliplatin (GEMOX regimen) could be an alternative treatment as front-line therapy in cisplatin-unfit patients of CCA. Currently, some clinical trials explore novel chemotherapeutic regimens or the addition of a third agent to improve curative effect.^8,28–32^ A lot of researches revealed tyrosine kinase inhibitors, selective HSP90 inhibitor ganetespib, ALK and ROS1 inhibitor ceritinib, MEK inhibition, and epigenetic therapies are promising targeted therapies in CCA patients.^5,33^ Besides, novel molecular targets have been identified by some clinical phase I studies in CCA patients because nearly 40% of genetic alterations of patients are possibly targetable,^5,33^ such as anti-mesothelin antibody–drug conjugate, CDK4/6 inhibitors, and PARP inhibitor.^5,34–37^ Furthermore, ICIs as monotherapy have been disappointing in advanced CCA patients. Therefore, ICIs are being tested in combination with chemotherapy or target therapy to increase the antitumor efficacy.^8,28–30,38,39^

Lenvatinib was approved by the FDA for the treatment of solid tumors such as radiation‐refractory differentiated thyroid cancer and HCC.^11,40,41^ A phase 2 study revealed lenvatinib as a second-line therapy in patients with CCA which showed 12% ORR and 46% DCR.^40^ In addition, Angélique Saint et al. reported an HCC-iCCA patient with a high expression of PD-L1 feature who received pembrolizumab and accomplished a complete response.^2^ The phase Ib Keynote-028 trial reported pembrolizumab in PD-L1-positive CCA patients which achieved 17% SD and 17% PR. The Keynote-158 trial reported 5.8% ORR with median PFS and OS of 2.0 months and 7.4 months.^10^ Thus, the efficacies of lenvatinib and pembrolizumab as single-agent therapy are not well established in CCA patients. Up to now, LEP was approved for the treatment of HCC, endometrial carcinoma, and renal cell cancer by the FDA.^11^ A single-arm study revealed that LEP in CCA patients who experienced progression from prior therapy reported 25% ORR and 40.5% CR with median PFS and OS of 4.9 months and 11.0 months, respectively.^12^ Zhang et al. showed that an advanced iCCA patient with a high TMB and high expression of PD-L1 feature achieved pathological complete response (pCR) through the combination of PD-1 inhibitors and lenvatinib.^42^ Besides, LEAP-005 (NCT03797326) demonstrated that LEP plus pembrolizumab in colorectal cancer patients with previously treated advanced non-MSI-H/pMMR showed median time of 10.6 months (range, 5.9–13.1) and 22% ORR.^43^ Therefore, LEP is a hopeful therapy for advanced iCCA patients, and there is a wide room to investigate the optimal treatment conditions in iCCA patients. However, an interesting point in our patient is the MSI status was stable and the TMB was determined as intermediate (7.1 mutations/Mb). This means there might be another biomarker of immunotherapy.

PD-L1, TMB, dMMR, and MSI-H are deemed efficacy-related biomarkers of immunotherapy.^44^ However, only 3% of CCA have reported a dMMR phenotype or high TMB, and their roles have not been systematically evaluated in iCCA patients.^42,45^ In this report, our patient was positive for the presence of EBER which is EBV-encoded RNA. Similar to PD-L1, TMB, dMMR, and MSI-H, EBV might be another biomarker of immunotherapy. EBV is the first identified human tumor virus that might participate in the development of nasopharyngeal carcinoma (NPC), gastric carcinoma (GC), and lots of lymphomas. EBV established latent infection in human through the balance between EBV and human immune system.^46^ A report explored increasing tumor-infiltrating B cells and CD8^+^ T cells in the tumor immune environment (TIME) of EBVaiCCA which means EBVaiCCA might have better therapeutic efficacy for immunotherapy. Not only that, the nonEBVaICC had the smallest TIME component, and the TIME component in EBVaICC lymphoepithelioma-like (LEL) was somewhat larger than in EBVaICC conventional type (CT) which might reflect different levels of antitumor immune responses. In addition, overexpression of PD-L1 in EBVaICC also demonstrated all subtypes might benefit from immunotherapy. In this case, consistent with the findings of that article, the EBVaICC patient showed a significant response to the therapy of Pembrolizumab plus Lenvatinib. Moreover, we propose that CT-type of EBVaICC can also produce an important response to LEP treatment.^45^ Some research also found that EBVaiCCA might upregulate the expression of PD-L1 which provided the theoretical basis for anti-PD-L1 therapy in EBVaiCCA patients.^46,47^ Besides, research showed that LEP in CCA patients with positive PD-L1 expression reported a longer median PFS (6.3 vs. 4.5 months, *p* = .005) and a prolonged median OS (20.7 vs. 8.4 months, *p* = .03).^12^ Therefore, clinical studies found that EBV is related to a lot of carcinomas and might be the biomarker of immunotherapy in some EBV-associated cancers.^45^ In a phase Ib clinical study, Pembrolizumab in NPC patients with positive EBV reported 18% ORR.^48^ NCT01592370 also showed a highly effective efficiency in treating EBV-positive lymphomas.^48^ A lot of trials such as NCT03257163, NCT0258949, and NCT02951091 are assessing response to anti-PD-L1 inhibitors in EBV-associated GC (EBVaGC) patients.^48,49^ However, to date, there is no research investigating the role of EBV as a biomarker in EBVaiCCA patients treated with anti-PD-L1 antibodies.

Here, we introduce the application of the receptor tyrosine kinase inhibitor lenvatinib in combination with the anti-PD-L1 drug Pembrolizumab for EBVaiCCA patients. Firstly, based on a single-arm study, LEP is a hopeful therapy for advanced CCA patients.^12^ Therefore, we chose the LEP combination for this iCCA patient after first-line chemotherapy failed which effectively prolonged the survival time of this patient. However, the clinical benefit of LEP for iCCA patients has not been known. Whether LEP could be approved still needs more examination with large-scale randomized trials in the future. Secondly, although NGS showed that this female patient has low TMB and MSI-S, this patient still has a significant response to anti-PD-L1 antibodies. This means EBV might be another predictive biomarker for immunotherapy in ICC. However, the function of EBV in predicting the efficiency of immunotherapy is complicated. Further studies with larger numbers of cases are still needed to support our conclusion.

For all we know, this is the first report on the application of lenvatinib in combination with Pembrolizumab for EBVaiCCA patients. LEP provides a glimmer of hope for advanced EBVaiCCA patients. Positive EBV in iCCA patients might be considered biomarkers for response to immunotherapy which breaks through the limitation of the traditional view. However, additional reliable research with larger groups is needed to define the efficacy of this treatment and the function of EBV. EBVaiCCA patients would be the next group to benefit from immunotherapy.

## Data Availability

All the data regarding the findings are available within the manuscript.
